# Xyloglucan for the treatment of acute diarrhea: results of a randomized, controlled, open-label, parallel group, multicentre, national clinical trial

**DOI:** 10.1186/s12876-015-0386-z

**Published:** 2015-10-30

**Authors:** Lucio Gnessi, Vladimir Bacarea, Marius Marusteri, Núria Piqué

**Affiliations:** 1 Department of Experimental Medicine, University of Rome “La Sapienza”, 00161 Rome, Italy; 2 Department of Epidemiology, University of Medicine & Pharmacy, Targu-Mures, Romania; 3 Department of Medical Informatics and Biostatistics, University of Medicine & Pharmacy, Targu-Mures, Romania; 4 Department of Microbiology and Parasitology, Pharmacy Faculty, Universitat de Barcelona, Diagonal Sud, Facultat de Farmàcia, Edifici A, Av Joan XXIII, 08028 Barcelona, Spain

**Keywords:** Acute diarrhea, Efficacy, Safety, Onset of action, Gelatin, Xyloglucan, Mucosal protectors, Stools, Abdominal pain, Vomiting

## Abstract

**Background:**

There is a strong rationale for the use of agents with film-forming protective properties, like xyloglucan, for the treatment of acute diarrhea. However, few data from clinical trials are available.

**Methods:**

A randomized, controlled, open-label, parallel group, multicentre, clinical trial was performed to evaluate the efficacy and safety of xyloglucan, in comparison with diosmectite and *Saccharomyces* in adult patients with acute diarrhea due to different causes.

Patients were randomized to receive a 3-day treatment. Symptoms (stools type, nausea, vomiting, abdominal pain and flatulence) were assessed by a self-administered *ad-hoc* questionnaire 1, 3, 6, 12, 24, 48 and 72 h following the first dose administration. Adverse events were also recorded.

**Results:**

A total of 150 patients (69.3 % women and 30.7 % men, mean age 47.3 ± 14.7 years) were included (*n* = 50 in each group). A faster onset of action was observed in the xyloglucan group compared with the diosmectite and *S. bouliardii* groups. At 6 h xyloglucan produced a statistically significant higher decrease in the mean number of type 6 and 7 stools compared with diosmectite (*p* = 0.031). Xyloglucan was the most efficient treatment in reducing the percentage of patients with nausea throughout the study period, particularly during the first hours (from 26 % at baseline to 4 % after 6 and 12 h). An important improvement of vomiting was observed in all three treatment groups.

Xyloglucan was more effective than diosmectite and *S. bouliardii* in reducing abdominal pain, with a constant improvement observed throughout the study. The clinical evolution of flatulence followed similar patterns in the three groups, with continuous improvement of the symptom. All treatments were well tolerated, without reported adverse events.

**Conclusions:**

Xyloglucan is a fast, efficacious and safe option for the treatment of acute diarrhea.

**Trial registration:**

EudraCT number 2014-001814-24 (date: 2014-04-28)

ISRCTN number: 90311828

## Background

According to the World Health Organization (WHO), acute diarrhea is defined as the production of three or more passages of loose or watery stools a day in a 24-h period, for less than 14 days [1, 2]. In non severe acute diarrhea of gastroenteritic origin, the stools do not contain visible amounts of blood or mucus [1].

Diarrhea is the expression of an altered homeostasis of the bowel [3], being intestinal infections (viral, bacterial and parasitic) the most common cause. Other causes include side effects of drugs (especially antibiotics), infections not associated with the gastrointestinal tract, food poisoning, and allergy [2, 4, 5].

Despite the introduction of oral rehydration therapies, annual global mortality rate is still high, with an estimate of 2.5 million people (with higher rates in children [5–7]. After perinatal death (23 %) and acute respiratory infection (18 %), acute diarrhea represents the third cause of death (15 %) in children under the age of 5 living in developing countries [5–7].

The recommended treatment for acute diarrhea consists of oral rehydration. Antibiotics, motility inhibitors such as loperamide, or substances that decrease water and electrolyte secretion such as racecadotril can be useful, although with some adverse events (such as the bacterial overgrowth induced by motility-reducing drugs), only in very specific situations [5].

Currently, there is a strong rationale for a new class of drugs, which may be defined as “mucosal protectors” although further studies are needed to completely assess the efficacy of these products [3, 8]. Xyloglucan, which form a bio-protective film is a good candidate in the treatment of acute diarrhea.

Xyloglucan, extracted from the seeds of the tamarind tree (*Tamarindus indica*), has recently received European approval for restoring the physiological functions of the intestinal walls. In the form of capsule form for adults and powder for paediatric use, xyloglucan has been specifically formulated for the control and reduction of symptoms related to diarrheal events of different aetiologies, such as abdominal tension and frequent emissions of faeces [9, 10].

Xyloglucan, if ingested in due amounts, stratify on the intestinal mucosa to form a bio-protective film that improves the resistance of the mucosa to pathologic aggressions and helps to restore its normal function. Xyloglucan has been shown to increase the Trans Epithelial Electrical Resistance (TEER), an index of good function of the mucosal tight junctions in cell cultures *in vitro*, confirming its ability to counteract mucosal permeability after leakage induced with *E. coli* exposure. This leakage of mucosal permeability is typical of diarrhea (Bueno et al., 2015; manuscripts in preparation). The same properties of xyloglucan have also been demonstrated *in vivo* by improving the mucosal leakage caused by intra-peritoneal injection of *E. coli* lipopolysaccharide (LPS - 1 mg/kg) or by intestinal exposure to cholera toxin (10 g/ml) in adult rats (Bueno et al., 2015; manuscripts in preparation). Gelatin, given with xyloglucan, like other proteins (i.e. vegetal proteins from pea), acts as a factor favouring a longer intestinal xyloglucan intestinal bioavailability prolonging the protective activity of xyloglucan.

Here we describe a randomized, multicenter, open-label study to assess efficacy, safety and time of onset of the antidiarrheal effect of xyloglucan, in comparison with two widely used anti-diarrheal products, *S. bouliardii*, containing the yeast probiotic *Saccharomyces boulardii*, and diosmectite, an absorbent activated natural aluminosilicate clay [1].

## Methods

The study protocol was approved by the Decision of the Ethical Commission for Scientific Research of the “Targu Mures” University of Medicine and Pharmacy with the no. 60 dated 8 July 2014 and procedures were in accordance with the ethical standards laid down in the Declaration of Helsinki, as revised in the year 2000. Written informed consent was obtained from all subjects. Patients were recruited in different Romanian private offices of general practitioners in the context of their routine clinical practice.

Inclusion criteria were a minimum age of 18 years and presence of acute diarrhea defined as the occurrence of >3 stools per day graded as 6 or 7 on the Bristol scale [11, 12]. Potential participants were excluded in case of allergy to one of the products´ ingredients, pregnancy or breastfeeding, recent surgery or serious and/or systemic diseases (such as inflammatory bowel disease). The use of oral rehydration solutions were not allowed during the study period.

The diagnosis was made according to the investigators’ judgment based on the clinical picture including objective (stools, vomiting and fever) and subjective symptoms (nausea, abdominal pain and bloating). Consistency of each stool was classified using the 7-point Bristol Stool Scale [11, 12].

The patients were randomly assigned to receive xyloglucan with gelatin, diosmectite and *S. bouliardii* at a ratio of 1:1:1. Xyloglucan-gelatin (Tasectan Plus^®^ Novintethical Pharma, SA) was administered in the form of oral capsules (containing xyloglucan, gelatin of porcine origin, corn starch and magnesium stearate). Diosmectite (Smecta^®^, Pharmaplan, SA) was administered as powder for oral solution (excipients: glucose monohydrate, saccharin sodium an orange-vanilla flavor). *S. bouliardii* was administered as oral capsules (Ultra-Levura^®^, Zambon, SA; excipients: lactose monohydrate and magnesium stearate).

During the first enrolment visit, patients from the 3 groups were given a 3-day treatment (two capsules every 6 h in the case of xyloglucan, three sachets/day in the case of diosmectite and 2 capsules/day in the case of *S. bouliardii*), with the first dose being administered at the time of recruitment (visit 1). Patients were instructed to return all packages of the used and unused product at the post-treatment visit (visit 2, 72 h after visit 1).

During visit 1, patients also received the patient’s daily diary, to assess the consistency of stools and the presence of diarrheal symptoms (both objective and subjective) at 1, 3, 6, 12, 24 and 48 h following the first dose administration. Stools emissions (including number of emissions/day), with mucus and/or blood, were recorded and consistency of each stool was assessed using the 7-point Bristol Stool Scale (type 1 corresponds to separate hard lumps, like nuts while type 7 corresponds to watery, no solid pieces, entirely liquid) [11, 12]. The presence of subjective symptoms such as nausea, vomiting (including number of vomits/day), abdominal pain and flatulence was also assessed.

At visit 2, which took place 72 h after visit 1, the investigators reviewed the patient’s daily diary. During this visit, a symptoms assessment was also performed by patient’s interview and exploration, to assess whether healing was achieved or treatment should be continued. All these data were transferred into the patient’s case report form (CRF).

The primary efficacy variable was the variation of the prevalence of diarrheal symptoms during the 3-day treatment and the rapidity of action of the studied products in the intention-to-treat (ITT) population, defined as all randomized patients who had at least one post-treatment measurement.

The primary safety variable was the prevalence of adverse events in the three groups of patients in the safety population, defined as all patients who received at least one dose of the studied product.

Sample size (*n* = 150, *n* = 50 in each group) was calculated to have a 80 % power to show, with 95 % probability, differences in the prevalence of diarrheal symptoms.

Descriptive analyses (within-patient n, mean, median, standard deviation, minimum and maximum) were performed for quantitative variables and frequency counts by category were calculated for qualitative variables. Following the results of normality tests (Kolmogorov-Smirnow test), data obtained at the two visits (baseline and at day 3) were compared by means of Friedman’s Anova and Kendall’s coefficient of concordance for non-parametric dependent data. Comparisons of data obtained at a certain visit among the three treatments were performed by means of Mann–Whitney U test for non-parametric and independent data. Two-sided *p*-values were obtained and statistically significant results were declared if *p* < 0.05.

## Results

**Fig. 1:**
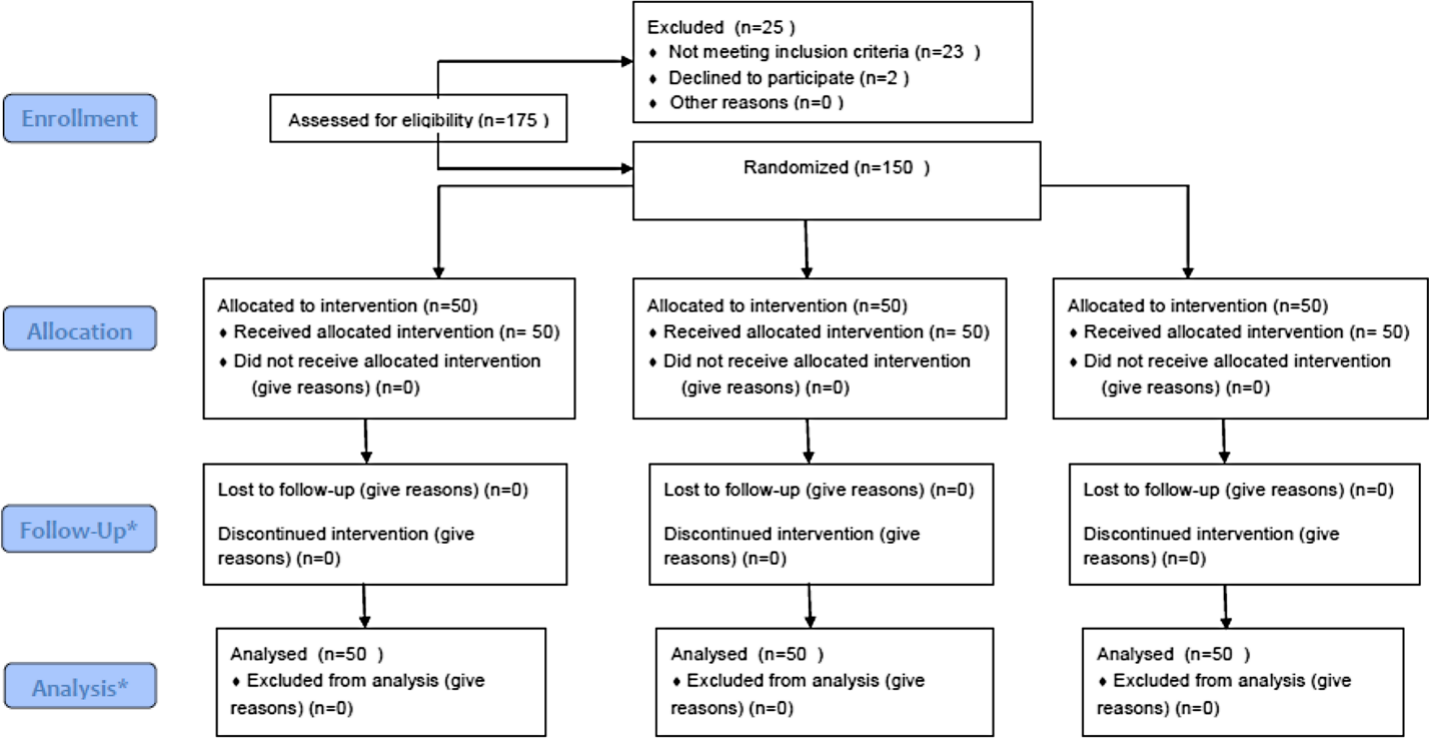
Study flow chart. *No subjects were Lost to Follow-up or Discontinued due to the short period of the study (72 hours)

A total of 150 patients were included in the study (50 in each group). All randomized patients had at least one post-treatment measurement and received all doses of the product, thus the ITT, PP and safety populations coincided (see flow chart in Fig. 1).

Table 1 shows the demographic characteristics of the patients. The groups were homogeneous, with a prevalence of women in the whole sample (69.3 % women vs 30.7 % men) and in all three groups and with a mean age of 47.3 years (Table 1).

**Table 1:** Demographic characteristics among groups

	Statistic variable	Xyloglucan	Diosmectite	*S. bouliardii*	Total	*p*-value
Gender (F/M)	n (%)	38 (76.0)/12 (24.0)	33 (66.0)/17 (34.0)	33 (66.0)/17 (34.0)	104 (69.3)/46 (30.7)	0.223
Age (years)	Mean (SD)	48.4 (14.5)	46.3 (16.9)	47.1 (12.5)	47.3 (14.7)	0.199

*F* female, *M* male, *SD* standard deviation

No statistically significant differences between treatment groups were noted regarding the demographic characteristics

**Fig. 2:**
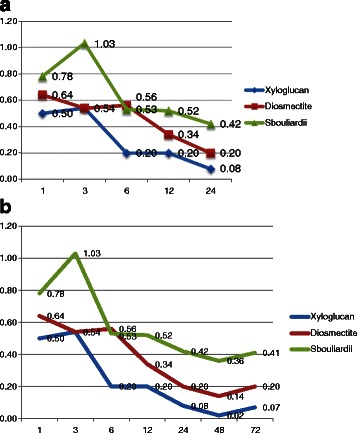
Clinical evolution of diarrheal symptoms (mean number of type 6 and 7 stools) among groups. **a** Mean number of type 6 and 7 stools during the first 24 h. **b** Mean number of type 6 and 7 stools during the study period

During the first 24 h of treatment, the xyloglucan group showed a faster onset of action and improvement of diarrheal symptoms, measured as mean number of type 6 and 7 stools, compared with the diosmectite and *S. bouliardii* groups. Accordingly, in the xyloglucan group the highest reduction of the number of type 6 and 7 stools was observed at 6 h with an effect that was statistically significant compared with diosmectite group (*p* = 0.031) (Table 2). The highest effect of diosmectite was observed at 12 and 24 h, although no statistically significant differences with xyloglucan group were found (Fig. 2). On the contrary, a higher efficacy was observed in xyloglucan group in comparison with *S. bouliardii* group at 12 and 24 h. At 48 and 72 h there were no statistically significant differences among the three groups in terms of efficacy (Fig. 2). As a general rule, both xyloglucan and diosmectite groups showed greater efficacy during the entire treatment period, compared with *S. bouliardii* (Table 2) (Fig. 2)

**Table 2:** Evolution of the mean number of dehydrating stools (type 6 and 7) during the study period

Time points	Xyloglucan	Diosmectite	*S. bouliardii*	*p*
1 h	0.50	0.64	0.78	X v D: 1.00
				X v S: 1.00
				D v S: 0.80
3 h	0.54	0.54	1.03	X v D: 1.00
				X v S: 0.99
				D v S: 1.00
6 h	0.20	0.56	0.53	X v D: 0.03
				X v S: 1.00
				D v S: 1.00
12 h	0.20	0.34	0.52	X v D: 0.70
				X v S: 1.00
				D v S: 0.52
24 h	0.08	0.20	0.42	X v D: 0.72
				X v S: 1.00
				D v S: 1.00
48 h	0.02	0.14	0.36	X v D: 1.00
				X v S: 1.00
				D v S: 1.00
72 h	0.07	0.20	0.41	X v D: 1.00
				X v S: 1.00
				D v S: 1.00

**Fig. 3:**
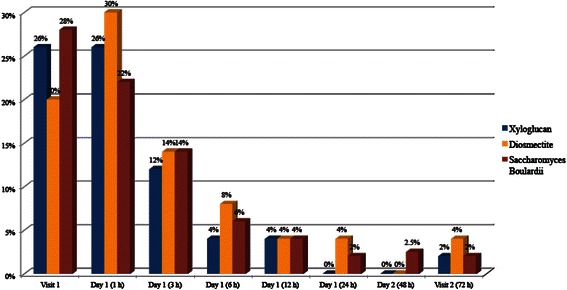
Percentage of patients with nausea during the study period

A higher efficacy of xyloglucan in reducing the percentage of patients with nausea was observed throughout the study period, particularly during the first hours. The percentage of patients with nausea progressively decreased starting from the beginning of xyloglucan treatment. 72 h after visit 1 only 2 % of patients treated with xyloglucan had nausea (Fig. 3). In the diosmectite group, the percentage of patients with nausea increased during the first hour post-administration, from 20 % at visit 1 to 30 %, and then decreased to 14 % at 3 h, 8 % after 6 h and 4 % after 12 and 24 h. At 48 h after visit 1, no patients had nausea, while at visit 2, a 4 % of patient had it (Fig. 3). Finally, in the group treated with *S. bouliardii*, the prevalence of nausea progressively decreased from 28 % at visit 1 to 2 % at visit 2 (Fig. 3).

**Fig. 4:**
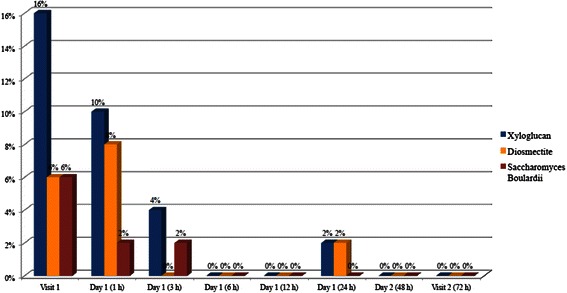
Percentage of patients with vomiting during the study period

An important improvement of vomiting was observed in all three treatment groups, with null percentages at 6 and 12 h after visit 1. At 24 h, 2 % of the patients in xyloglucan group and diosmectite group had vomiting and no patients had vomiting at this time point in the *S. bouliardii* group (Fig. 4).

**Fig. 5:**
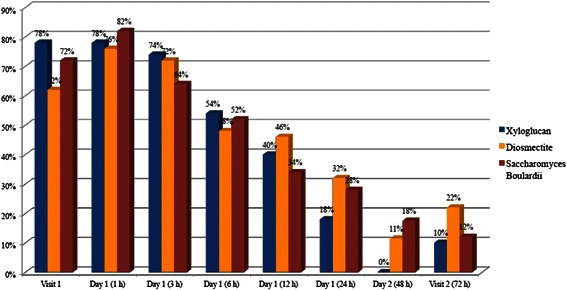
Percentage of patients with abdominal pain during the study period

Xyloglucan was more effective than diosmectite and *S. bouliardii* in reducing abdominal pain, with a constant improvement observed throughout the study period. In the groups treated with diosmectite and *S. bouliardii*, an increase in the percentage of patients with abdominal pain was observed within the first hour post-visit 1. At visit 2, the lowest percentage of patients with abdominal pain was recorded in xyloglucan group (10 %), in comparison with diosmectite (22 %) and *S. bouliardii* (12 %) groups (Fig. 5).

**Fig. 6:**
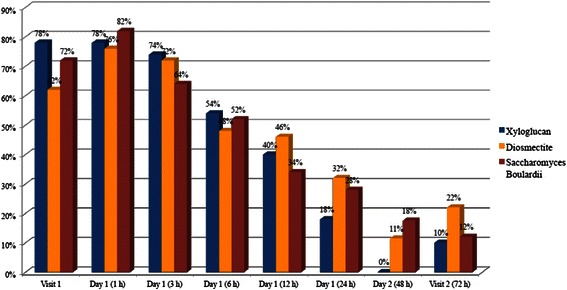
Percentage of patients with flatulence during the study period

The evolution of flatulence followed similar patterns in the three groups, with a slight worsening during the first hour after visit 1 and continuous improvement until visit 2. The greatest improvement was reported in patients of xyloglucan group, with 10 % of patients with flatulence at visit 2, compared with diosmectite (30 %) and *S. bouliardii* (18 %) groups (Fig. 6).

Adherence was 100 % in the 3 groups.

All treatments were safe and well tolerated, with no adverse reaction being reported during the study.

## Discussion

The emergence of new protective agents of the intestinal mucosa with improved film forming efficacy, such as xyloglucan, offers new alternatives for a more efficient control of diarrheal diseases [3, 13].

In the present study, we assessed the efficacy and safety of xyloglucan, in comparison with diosmectite and *S. bouliardii,* widely used products for the treatment of acute diarrhea. These results are also in line with other studies with xyloglucan, in comparison with oral rehydration solution [9].

We found that xyloglucan is efficacious to rapidly improve the main symptoms of acute diarrhea, equal or better than diosmectite and *S. bouliardii* [14–16].

One of the main findings of this study is the faster onset of action observed in the xyloglucan group compared with diosmectite and *S. bouliardii*.

Such a rapid onset of action is particularly desirable in the treatment of acute nonspecific diarrhea [17], to decrease severity of symptoms, dehydration and risk of transmission in case of diarrhea of infectious origin [18].

We also observed a rapid effect of xyloglucan in reducing vomiting, another important symptom causing dehydration [19]. It should be noted that prevalence of vomiting at visit 1 was higher in the xyloglucan group than in the rest of groups. However, during the first 3 h, we observed in the xyloglucan group, a marked sustained decreased in the percentage of patients with this symptoms. In the other groups, although initial prevalence was lower, this sustained decreased during the first 3 h was not observed as clear as in the xyloglucan group.

Also of relevance is the reduction of abdominal pain observed in patients treated with xyloglucan, in comparison with diosmectite and *S. bouliardii*, probably due to the direct protective effect xyloglucan on the intestinal wall, which reduces the exposure of intestinal cells to inflammatory agents that trigger abdominal pain. This mechanism of action can explain the differences observed with the probiotic *S. bouliardii*, whose effect on diarrhea is thought to be related to competition with pathogenic microorganisms for nutrients on adhesion sites, and possibly by secretion of probiotic compoundsthat inhibit the growth of pathogenic microorganisms [1]. As already reported [1], the efficacy of this probiotic in acute diarrhea has been related to stools consistency. Rapid reduction of abdominal pain is relevant in the control of diarrhea, increasing patient’s quality of life and assuring a rapid recovery [20]. It is also of relevance the better effect of xyloglucan, in comparison with diosmectite, an adsorbent clay mineral with coating protective properties [1].

Reduction of abdominal pain is also important for the whole management of diarrhea, in which, the administration of anti-inflammatory drugs as NSAIDs can alter the integrity of mucus layer [1, 2, 4, 5].

Finally, as expected, all three treatments reduced flatulence, a symptom that importantly decreases the patient’s quality of life. In the case of gelatin and xyloglucan, the formation of the biofilm could reduce bacterial proliferation and the formation of gas, although further preclinical studies should be performed to demonstrate it. In the case of diosmectite, it has been found that diosmectite can reduce the production of hydrogen in the colon during microbial fermentation [1, 21].

The present study is part of the development of new food supplements that contain mucosal protectors, with film-forming properties. In the case of xyloglucan, we have demonstrated its properties in preclinical *in vitro* and *in vivo* models. This study is part of the clinical development of xyloglucan, and the results obtained are also in line with those obtained in another study in children with acute gastroenteritis [22]. As future research, other studies could be done to assess the effect of xyloglucan in other gastrointestinal diseases associated with diarrheal symptoms.

## Conclusions

In conclusion the administration of xyloglucan is an efficacious and safe option in the clinical practice for the treatment of acute diarrhea, with a rapid onset of action in reducing diarrheal symptoms.

## Abbreviations

WHO: World Health Organization
TEER: Trans Epithelial Electrical Resistance
LPS: Lipopolysaccharide
CRF: Case report form
ITT: Intention-to-treat
NSAIDs: Non-steroidal anti-inflammatory drugs
